# Apolipoprotein and sphingolipid measurements: Can be used in the clinical practice of atrial fibrillation diagnosing and evaluating the cryoablation effectiveness?

**DOI:** 10.1371/journal.pone.0315905

**Published:** 2025-03-04

**Authors:** Patrycja Bielawiec, Ewa Harasim-Symbor, Karolina Gołaszewska, Adrian Chabowski, Katarzyna Hodun, Klaudia Sztolsztener

**Affiliations:** 1 Department of Physiology, Medical University of Bialystok, Białystok, Poland; 2 Department of Cardiology, Ministry of Interior and Administration Hospital in Bialystok, Białystok, Poland; Universite Paris Diderot-Paris7 - Batiment des Grands Moulins, FRANCE

## Abstract

Atrial fibrillation (AF) has become the most common arrhythmia of clinical importance. A well-established and recommended therapeutic option for AF is the balloon-based cryoablation (CBA) method. There are still no sensitive biomarkers for AF prediction and cryoablation effectiveness assessment, therefore in our prospective study, we examined the plasma content of apolipoproteins (Apo) and sphingolipids, as well as the distribution of selected sphingolipids among lipoprotein fractions. The study included 33 patients with AF on admission and 24 h after cryoablation therapy, while 20 healthy volunteers were recruited to the control group. Plasma Apo concentrations were determined using a multiplex assay kit measuring fluorescence signal, whereas the high-performance liquid chromatography (HPLC) method was applied to assess the total plasma sphingolipid levels as well as their content in isolated lipoprotein fractions. Our results showed that cryoballoon ablation in AF patients markedly reduced the level of almost all Apo compared to the individuals from the control and Pre-CBA groups (Apo-A1: −25.9% and −20.0%, Apo-A2: −19.9% and −17.3%, Apo-B: −26.8% and −14.4%, Apo-C1: −20.3% and −13.4%, Apo-D: −15.9% and −22.2%, Apo-E: −18.3% and −14.3%, and Apo-J: −36.4% and −21.5%, p < 0.05, respectively). Importantly, the area under the curve of Apo-J (AUC 0.81; 95% CI, 0.71–0.92) indicates that it might be a useful biomarker of cryotherapy success in AF patients. Moreover, we also observed a pronounced increase in sphinganine (Sa; +33.5%), sphingosine (So; +24.6%), sphinganine-1-phosphate (Sa1P; +34.3%), and sphingosine-1-phosphate (So1P; +22.3%) concentrations in the Pre-CBA group in comparison with controls. This is the first study that evaluates such a broad panel of Apo and sphingolipids in patients with AF undergoing the CBA procedure, however, to confirm whether any of these parameters could be a clinically useful biomarker for predicting AF or assessing the effectiveness of treatment, further research will be necessary due to limitations of the study.

## Introduction

Currently, atrial fibrillation (AF) is the most frequently diagnosed cardiac arrhythmia in clinical practice worldwide [1]. AF, defined as supraventricular tachyarrhythmia, is a risk factor for systemic embolism, stroke, and heart failure and is associated with significant cardiovascular and overall mortality [2]. Epidemiological studies have shown that the increase in the incidence of AF is correlated with age, which is a prominent risk factor, however, a growing burden of other comorbidities, including hypertension (HT), coronary artery disease (CAD), obesity, and type 2 diabetes mellitus (T2DM), has been shown to favor the development of cardiac arrhythmias [3]. In recent years, significant advances have been made in AF treatment, and according to the European Society of Cardiology (ESC) guidelines, the well-established method of pulmonary vein isolation (PVI) is used in patients with AF who are unresponsive or intolerant of antiarrhythmic drugs [1]. The form of PVI with cryoballoon catheter ablation (CBA) has proven to be an effective and well-tolerated method of AF treatment [4]. However, despite technical and medical progress in ablation therapy, predicting the risk of the recurrence of cardiac arrhythmia in patients after treatment remains difficult. Therefore, it seems important to search for sensitive biomarkers for early detection of AF and cryoablation efficacy assessment.

Studies in recent years have shown that obesity and accompanying dyslipidemia are important risk factors predisposing to the onset of cardiovascular diseases (CVDs), including atherosclerosis and CAD, which are closely associated with an increased risk of AF. The most common lipid abnormalities found in patients with CVDs include high triacylglycerols (TAG), elevated low-density lipoprotein cholesterol (LDL-C) levels, and decreased numbers of high-density lipoprotein cholesterol (HDL-C) particles [5]. Lipoproteins are molecules that transport dietary lipids in the bloodstream, mainly cholesterol (Chol) and TAG [6]. They are comprised of a hydrophobic center (containing predominantly Chol and TAG), phospholipids (PL), and apolipoproteins (Apo) embedded in the outer shell of the particle [7]. Plasma lipoproteins are classified into several groups based on their relative lipid content, size, and the presence of a major specific protein - Apo, which gives them a functional identity that determines their role [8]. Today, it is unclear whether plasma lipoprotein parameters other than LDL-C and HDL-C could provide clinically relevant prognostic information about cardiovascular risk, give more data about therapy efficacy, or specify more appropriate treatment goals, especially in patients with AF. Therefore, in recent years, more and more attention has been paid to Apo, which plays a crucial role in lipid transport and metabolism, thus these molecules can significantly affect the whole body’s lipid balance [6]. Numerous studies have proven that Apo differ in their biological functions, which is of significant clinical importance. Apolipoprotein A1 (Apo-A1) is the primary structural protein of HDL-C particles, and its elevated level is correlated with a lower risk of atherosclerosis [9,10]. Conversely, apolipoprotein A2 (Apo-A2) although it is the second most abundant protein in HDL-C, is associated with proatherogenic properties and is a strong predicting factor of cardiovascular risk [8]. Another protein that plays an important role in lipid clearance is apolipoprotein B (Apo-B), which is part of atherogenic lipoproteins, such as LDL-C, intermediate-density lipoprotein cholesterol (IDL-C), and very low-density lipoprotein cholesterol (VLDL-C) particles, and its function is to transport lipids from the liver and intestines to peripheral tissues [11]. Moreover, the group of C apolipoproteins (Apo-C1 and Apo-C3) is involved in the regulation of hepatic uptake of triglyceride-rich lipoproteins (TRL; chylomicrons and VLDL-C) by inhibiting the activity of lipoprotein lipase (LPL), which may result in hypertriglyceridemia [12]. Besides, both Apo-C1 and Apo-C3 impair the plasma TAG clearance, recent studies indicated their opposite impact on HDL-C functionality where Apo-C1 has been shown to have positive effects on HDL-C atheroprotective functions, whereas Apo-C3 exhibits negative effects [13,14]. Other molecules involved in the transport and metabolism of lipophilic particles are apolipoproteins D (Apo-D) and E (Apo-E). Several studies have shown that they have cardioprotective effects related to these proteins’ antioxidant and antiatherogenic properties, respectively [15,16]. In contrast, a growing body of evidence indicated a positive correlation between elevated plasma apolipoprotein H (Apo-H; also known as β2-glycoprotein I) and apolipoprotein J (Apo-J; also referred to as clusterin) concentrations and the incidence of metabolic syndrome and associated systemic inflammation [17,18]. It is interesting to note that Apo-H is also implicated in various thrombotic processes as it inhibits the activity of the platelet prothrombinase as well as adenosine diphosphate (ADP)-induced platelet aggregation [19]. Disturbances in the concentration and composition of lipoproteins, with particular emphasis on Apo level, may result in dysregulation of the transport and metabolism of dietary fats, leading to dyslipidemia, which is a significant risk factor for the onset of AF.

Other molecules that have been found to be associated with plasma lipoproteins are sphingolipids. They serve as biologically active structural lipid compounds of cell membranes and are also recognized as signaling molecules participating in essential cellular processes including proliferation, apoptosis, and immune pathways [20]. The most important representatives of the sphingolipid class are sphingomyelin (SM), sphinganine (Sa; d18:0), sphingosine (So; d18:1), sphingosine-1-phosphate (So1P; d18:1P), as well as ceramide (Cer; d18:1 Cer) [21]. In recent years, Cer and So1P have become the subject of many investigations since those bioactive sphingolipids critically modulate a myriad of cellular biological processes [20]. A growing body of research focusing on Cer has shown that it acts as a second messenger, regulating cell growth, migration as well as processes of proliferation and apoptosis [22]. Furthermore, Cer is a precursor for other sphingolipids such as So1P, which has well-documented anti-apoptotic and mitogenic properties and plays an essential role in cardioprotection [23]. Due to the multidirectional action of sphingolipids, the dysregulation of their metabolism may be considered as one of the risk factors for the development of CVDs, including AF. Hence, it is suggested that the content of particular sphingolipids may serve as useful biomarkers in the prognosis of cardiac events in clinical practice, possible indicators of treatment effectiveness, or a new therapeutic target in the course of AF therapy.

AF is a significant burden for patients, social and economic health worldwide. In recent years, despite a significant expansion of knowledge about the pathophysiology of AF, there is still a lack of data on the effective management of this disease. Therefore, the purpose of this prospective study was to investigate the plasma concentration of a broad panel of Apo as well as sphingolipids levels with simultaneous evaluation of selected sphingolipids distribution in individual lipoprotein fractions, which could help to facilitate targeting screening programs for early detection of AF and assessment of therapy success in CBA-treated patients.

## Materials and methods

### Study population

Thirty-three patients with refractory AF, which were either intolerant to antiarrhythmic medication or resistant to treatment were involved in the study and qualified for PVI using the cryoballoon ablation technique. The individuals with acute coronary syndrome, coronarography/electrical cardioversion, decompensated heart failure, moderate to severe valvular heart disease, renal failure (serum creatinine concentration > 2 mg/dl), hyperthyroidism, and active infection within the last six months in the history were excluded from the study. The clinical characteristics (age, gender, BMI as well as accompanying comorbidities) of patients with AF were presented in our previous publication [24].

The reference group consisted of twenty healthy volunteers. All participants were carefully examined and included in the Control group based on the following criteria: ≥ 40 years of age, good health, the lack of lipid-lowering or antihypertensive therapies, and cardiovascular diseases in the history.

### Ethics statement

The study was approved by the Bioethics Committee of the Medical University of Bialystok, Poland (approval number: R-I-002/308/2018) and performed following the recommendations of the Declaration of Helsinki. All participants from the study and reference groups were recruited between July 2018 and August 2019 and confirmed the written informed consent to participate in the study and to collect and store blood samples.

### Ablation procedure

For the ablation procedure, diagnostic electrophysiology (EP) catheters were used. One of them, a diagnostic decapolar electrode was positioned in the coronary sinus, and the other, a quadripolar electrode was positioned in the right atrium. After the appropriate placement of EP catheters, a single transseptal catheterization was performed. AF-patients were subjected to PV isolation by applying a cryoballoon catheter (28 mm, Arctic Front Advance; Medtronic, Inc., Minneapolis, MN, USA) embed by the FlexCath advance steerable sheath 12F (Medtronic Inc., Minneapolis, MN, USA). Optimal vessel occlusion has been visualized by the administration of selective contrast. The procedure of cryothermal applications persevered for 3 min. To stimulate the right phrenic nerve, a quadripolar electrode from the superior vena cava was used. Moreover, an additional freeze was performed for the PV isolation failure. The lack of PV activity after the application was considered as a PVI. After the CBA procedure, we conducted a regular follow-up consisting of consultation 3 and 6 months after the cryoapplication, which included evaluation of 12-lead electrocardiogram (ECG) or 24-hour Holter monitoring in an outpatient clinic and a detailed history of symptoms related to the arrhythmia (chest discomfort, dizziness, palpitations, and fatigue) (for all procedural and postprocedural details of the cryoablation see our previous publication [24]).

### Blood samples collection

Blood samples from patients with AF were taken before the ablation procedure (pre-CBA) and 24 h after catheter ablation (24 h post-CBA). Blood samples from healthy individuals were taken only once. Blood from patients was collected in tubes containing EDTA as an anticoagulant. Within 30 min after collection, samples were centrifuged at 2000 × g for 15 min at 4 °C, and then the obtained plasma samples were placed in new tubes and stored at −80 °C until further determinations.

### Multiplex immunoassay

Based on the multiplexing method with sensitive covalently coupled magnetic beads, an immunoassay kit (Bio-Plex Pro Human Apolipoprotein 10-plex assay; Bio-Rad, Hercules, CA, USA) was used to determine the level of selected apolipoproteins, as follows: Apo-A1, Apo-A2, Apo-B, Apo-C1, Apo-C3, Apo-D, Apo-E, Apo-H, and Apo-J. In accordance with the protocol described by the manufacturer, samples of plasma were centrifuged at 1000 × g for 15 min at 4 °C to remove particulates and then three times diluted (1:50000) using sample dilution buffer (Bio-Rad, Hercules, CA, USA). The blank, standards, and diluted samples were applied to the appropriate well of the microplate, and then the capture beads were added to each well. After an incubation series of washes, the detection biotinylated antibodies were added following the next incubation. The obtained complex was exposed to add the solution of streptavidin-phycoerythrin (SA-PE) and resuspended beads. In the end, the internal fluorescence was detected and the signal intensity was measured by Bio-Plex 200 Reader System (Bio-Rad, Hercules, CA, USA). The obtained signals expressed as median fluorescence intensity (MFI) were used to plot standard curves and finally to calculate the concentration of individual apolipoproteins. The datasets of apolipoproteins concentrations obtained in our study are provided in the Supporting Information file S1 Data.

### Isolation of lipoproteins

Selected lipoproteins were isolated from the platelet-free plasma using a modified version of the protocol reported in detail by Havel et al. [25]. The sequential flotation ultracentrifugation of plasma in NaBr solution using an ultracentrifuge (Sorvall RC M120 GX, Thermo Scientific, Waltham, MA) equipped with a S120-AT2 rotor was performed. The EDTA (0.3 mM) was present in each preparation step to avoid oxidation of lipoproteins. Firstly, plasma was centrifuged at 120,000 rpm for 85 min at a density of 1.006 g/mL to obtain VLDL-C, next for 155 min at a density of 1.063 g/mL to obtain LDL-C, and for 260 min at a density of 1.21 g/mL to obtain HDL-C. After the final centrifugation step, the lipoprotein-depleted plasma containing albumin was recovered from the bottom of the tube. All centrifugations were conducted at 8 °C. A digital densitometer (Mettler Toledo, Columbus, OH) was used to verify the density of NaBr solutions used for density adjustments (Mettler Toledo, Columbus, OH). Before further analysis, the samples were kept at −80 °C.

### High-performance liquid chromatography method

In plasma and isolated lipoprotein fractions, the content of Sa (d18:0), So (d18:1), sphinganine-1-phosphate (Sa1P; d18:0P), So1P (d18:1P), and Cer (d18:1 Cer) was determined by the HPLC method according to the previously described protocol by Min et al. [26]. Briefly, 250 μl of plasma samples were homogenized with ultrasonication in ice-cold water. Then, with the addition of internal standards (30 pmol C17-sphingosine-1-phosphate and 10 pmol of C17-sphingosine; Avanti Polar Lipids, Inc.; Alabaster, AL, USA) and acidified methanol, lipids from obtained homogenates were extracted by adding chloroform, 1 M NaCl, and 3 N NaOH. The upper alkaline aqueous phase containing Sa1P and So1P was then transferred into a new tube. The residual phosphorylated sphingoid bases in the chloroform phase were re-extracted twice with a 1:1 (v/v) solution of methanol and 1 M NaCl, and then all the aqueous fractions were combined. Next, into this combined solution we added alkaline phosphatase (Sigma Aldrich; Saint Louis, MO, USA), in order to dephosphorylate the sphingoid base-1-phosphates into sphinganine and sphingosine, which will enable indirect determination of their amount. To improve the extraction yield of released So, some chloroform was carefully placed at the bottom of this reaction tube. In the next step, the primary chloroform fractions containing free sphinganine and sphingosine as well as tubes with dephosphorylated sphingoid bases were washed with alkaline water and subsequently evaporated under a nitrogen stream. After that, the dried lipid residues were re-dissolved in ethanol and converted to their *o*-phthalaldehyde derivatives, which were transferred into separate inserts and determined by the use of HPLC system (Agilent Technologies; Santa Clara, CA, USA) equipped with a C18 reversed-phase column Omnisphere 5 (4.6 × 150 mm; Varian Inc., Lake Forest, CA, USA) and fluorescence detector.

Simultaneously, a small aliquot (50 μl) of the chloroform phase containing extracted lipids was taken from the primary tube and then was placed in a fresh tube containing C17-base (N-palmitoyl-D-erythro-sphingosine) as an internal standard.. Next, the samples were evaporated in a stream of nitrogen and then were subjected to alkaline hydrolysis in 1 M KOH in 90% methanol and heated for 60 minutes at 90 °C to deacylate ceramide into sphingosine. After that, the amount of sphingosine liberated from Cer was determined by means of HPLC as described above. Since the chloroform extract used for the Cer assay contains trace amounts of free sphingoid bases, the Cer concentration was corrected for the content of free sphingosine determined in the same sample. The datasets of sphingolipids concentrations obtained in our study are provided in the Supporting Information file S2 Data.

In brief, the HPLC technique was validated to ensure reliable and repeatable results in accordance with the International Council for Harmonization (ICH) guidelines. Peak height analyses included the measurement of the signal-to-noise ratio (S/N), with a threshold of three for the limit of detection (LOD) and an S/N > 10 for the limit of quantification (LOQ). For HPLC procedures, a blank and a placebo extract were applied as reference materials, known as a “cocktail” and retention marker solution, to ensure precise results in evaluating an analyte in the sample; the synthetic precursors, enantiomers, and excipients were applied to enhance the method’s selectivity. The standard deviation (SD) or relative standard deviation (RSD) was calculated from a suitable number of homogenous sample aliquots to establish a reliable technique. Multiple injections, at least five replicates, of the same reference solution were used to evaluate the precision, and the acceptable peak area precision value was required for the quantitative analysis of this method [27]. The average between-run variations (%CV) for the selected plasma sphingolipids measured by the aforementioned method are as follows: Sa 10.7%, So 3.9%, Sa1P 9.1%, So1P 5.8%, and Cer 0.8%. The within-run variations are as follows: Sa 9.8%, So 5.0%, Sa1P 8.2%, So1P 5.2%, and Cer 4.1%. The linear dynamic range of the above method for Sa and So is from 3 to 2000 pmol/ml of plasma, for Sa1P and So1P is from 6 to 2000 pmol/ml, and for Cer it is from 150 to 30,000 pmol/ml. The limit of detection in plasma is approximately 1 pmol/ml for Sa and So, approximately 2 pmol/ml for Sa1P and So1P, and approximately 30 pmol/ml for Cer [28].

### Statistical analysis

The data are presented as mean ± SD. The statistical analyses were carried out in GraphPad Prism 8.2.1. Software, Inc. (San Diego, CA, USA). The Shapiro-Wilk test was used to assess the normality distribution of datasets. The statistical differences were identified by one-way analysis of variance (ANOVA) followed by the non-parametric Mann-Whitney U test or the parametric *t*-test for variables with non-normality or normality distribution, respectively. The unpaired *t*-test was used to compare the results between two independent groups, i.e., Pre-CBA and 24 h post-CBA vs. Control group. The paired *t*-test was used to assess the differences between two paired measurements, i.e., 24 h post-CBA vs. Pre-CBA. The quantity determination of diagnostic values was performed by the ROC (receiver operating characteristic) curve analysis. For evaluation of the prognostic potential of apolipoproteins and sphingolipids, the ROC curve analysis were performed with the standard parameter using a hybrid Wilson-Brown method for 95% confidence interval (95% CI) calculation. This procedure was used to compare ROC curves for the two unpaired (Pre-CBA vs. Control and 24 h post-CBA vs. Control) or two paired (24 h post-CBA vs. Pre-CBA) samples and after that, the comparisons in the entire groups were used to generate AUC graphs. The hybrid Wilson-Brown method was applied to create a dot plot ROC curve graph for each pair of data, which approximated the area under the ROC curve by dividing it with vertical and horizontal lines. The ROC curve results were expressed as fraction values, which ranged from 0 to 1 [29–31]. The statistically significant was considered as a *p*-value < 0.05.

## Results

### The comparison of apolipoprotein concentrations in plasma of patients with atrial fibrillation (before and 24 h after cryoballoon ablation (CBA) therapy) and control individuals

**Fig 1:**
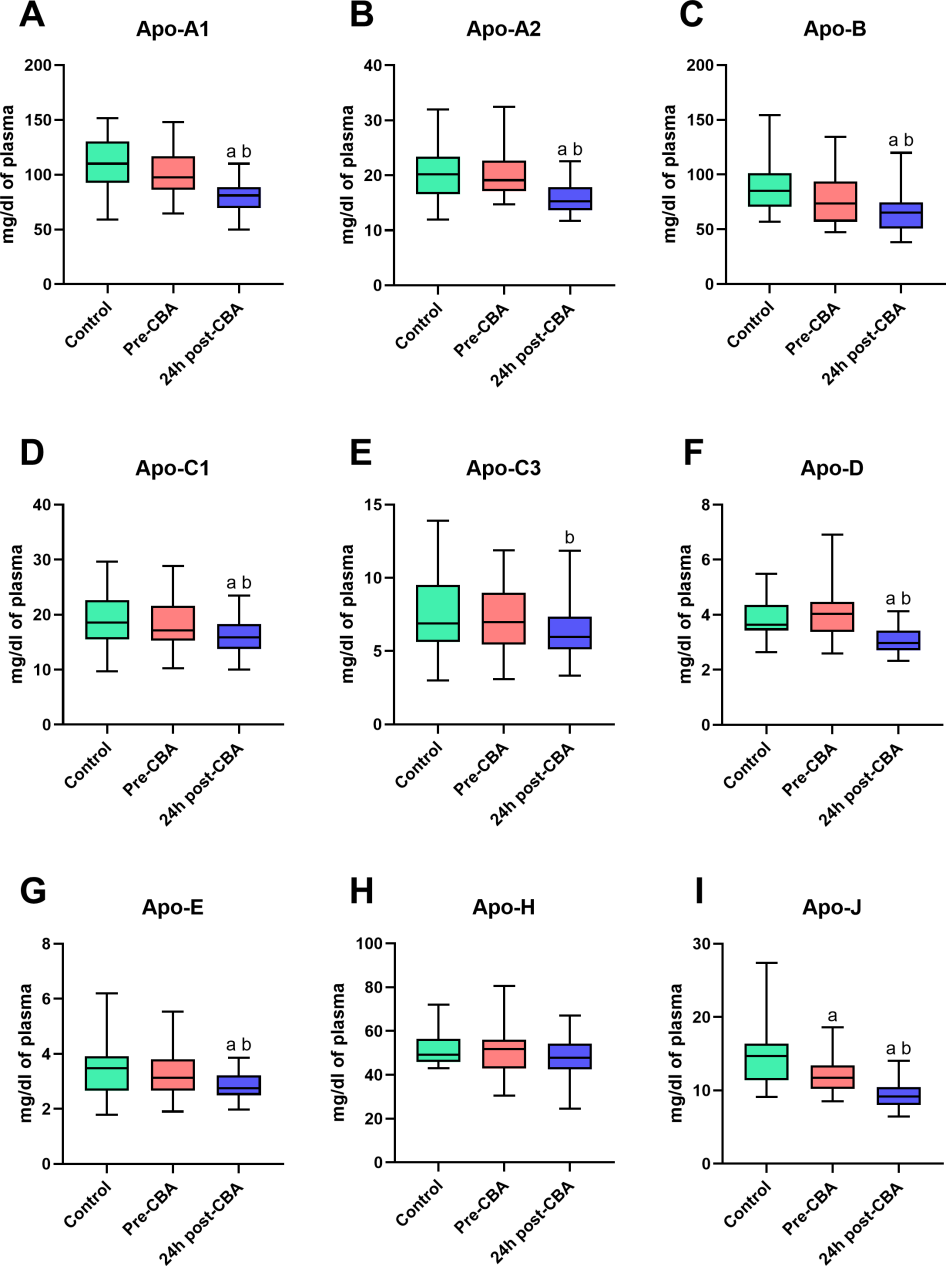
Apolipoproteins profile in patients with atrial fibrillation before (pre-CBA) and 24 h after (24 h post-CBA) cryoballoon ablation (CBA) therapy and healthy volunteers (Control) in plasma fraction. Apolipoprotein A1, A2, B, C1, C3, D, E, H and J (Apo-A1, -A2, -B, -C1, -C3, -D, -E, -H and -J). Data based on 20 independent measurements for the Control group and 29 independent measurements for both the Pre-CBA and 24 h post-CBA groups. The statistically significant difference was considered as a p-value < 0.05.

The patients with AF before CBA had a lower concentration of Apo-J in relation to the Control group (Apo-J: −19.0%; p < 0.05; Fig 1I). Additionally, a significantly decreased concentration of selected apolipoproteins in patients 24 h after CBA was observed compared to the healthy volunteers (Apo-A1: 25.9%; Apo-A2: −19.9%; Apo-B: −26.8%; Apo-C1: −20.3%; Apo-D: −15.9%; Apo-E: −18.3%; Apo-J: −36.4%; p < 0.05; Fig 1A,1B,1C,1D,1F,1G, and 1I). We also noticed that in patients with AF, the level of almost the same above-mentioned apolipoproteins was abolished 24 h after cryoballoon ablation than in the individuals from Pre-CBA group (Apo-A1: −20.0%; Apo-A2: −17.3%; Apo-B: −14.4%; Apo-C1: −13.3%; Apo-C3: −12.6%; Apo-D: −22.2%; Apo-E: −14.3%; Apo-J: −21.5%; p < 0.05; Fig 1A,1B,1C,1D,1E,1F,1G, and 1I).

The area under the ROC curve (AUC) values of Apo-A1, Apo-A2, Apo-B, Apo-C1, Apo-D, Apo-E, and Apo-J concentrations in patients 24 h after CBA in comparison with individuals from Control and Pre-CBA groups were significantly increased (Apo-A1: 0.81 and 0.78, Apo-A2: 0.80 and 0.80, Apo-B: 0.79 and 0.66, Apo-C1: 0.70 and 0.68, Apo-D: 0.80 and 0.84, Apo-E: 0.70 and 0.68, Apo-J: 0.89 and 0.81, respectively; p < 0.05; Table 1). Moreover, the AUC values of Apo-J in patients before CBA compared to the healthy volunteers were below 0.80 (0.72; p < 0.05; Table 1).

**Table 1:** The analysis of AUC values of apolipoprotein concentrations of patients with atrial fibrillation (before and 24 h after cryoballoon ablation (CBA) therapy) and control individuals in plasma fraction.

	The values of Area Under the ROC Curve, 95% Confidence Interval of AUC, and *p*-value		
	Pre-CBA vs. Control	24 h post-CBA vs. Control	24 h post-CBA vs. Pre-CBA
Apo-A1	0.61 0.44–0.77 0.2036	0.81 0.66–0.95 0.0003	0.78 0.66–0.90 0.0003
Apo-A2	0.55 0.38–0.72 0.5283	0.80 0.66–0.94 0.0004	0.80 0.69–0.91 0.0001
Apo-B	0.65 0.50–0.81 0.0718	0.79 0.66–0.91 0.0007	0.66 0.51–0.80 0.0416
Apo-C1	0.54 0.37–0.71 0.6353	0.70 0.54–0.86 0.0210	0.68 0.54–0.82 0.0193
Apo-C3	0.53 0.36–0.70 0.7295	0.64 0.74–0.80 0.1081	0.62 0.47–0.76 0.1237
Apo-D	0.57 0.40–0.73 0.4276	0.80 0.67–0.93 0.0004	0.84 0.74–0.94 < 0.0001
Apo-E	0.52 0.35–0.69 0.7993	0.70 0.54–0.86 0.0199	0.68 0.54–0.82 0.0177
Apo-H	0.54 0.37–0.70 0.6504	0.63 0.48–0.79 0.1213	0.59 0.44–0.74 0.2435
Apo-J	0.72 0.56–0.87 0.0101	0.89 0.80–0.98 < 0.0001	0.81 0.71–0.92 < 0.0001

Apolipoprotein A1, A2, B, C1, C3, D, E, H and J (Apo-A1, -A2, -B, -C1, -C3, -D, -E, -H and -J). The values of the Area Under the ROC Curve and 95% Confidence Interval of AUC are expressed in the range 0–1. Data based on 20 independent measurements for the Control group and 29 independent measurements for both the Pre-CBA and 24 h post-CBA groups. The statistically significant difference was considered as a p-value < 0.05.

### The comparison of sphingolipid concentrations in plasma of patients with atrial fibrillation (before and 24 h after cryoballoon ablation (CBA) therapy) and control individuals

**Fig 2:**
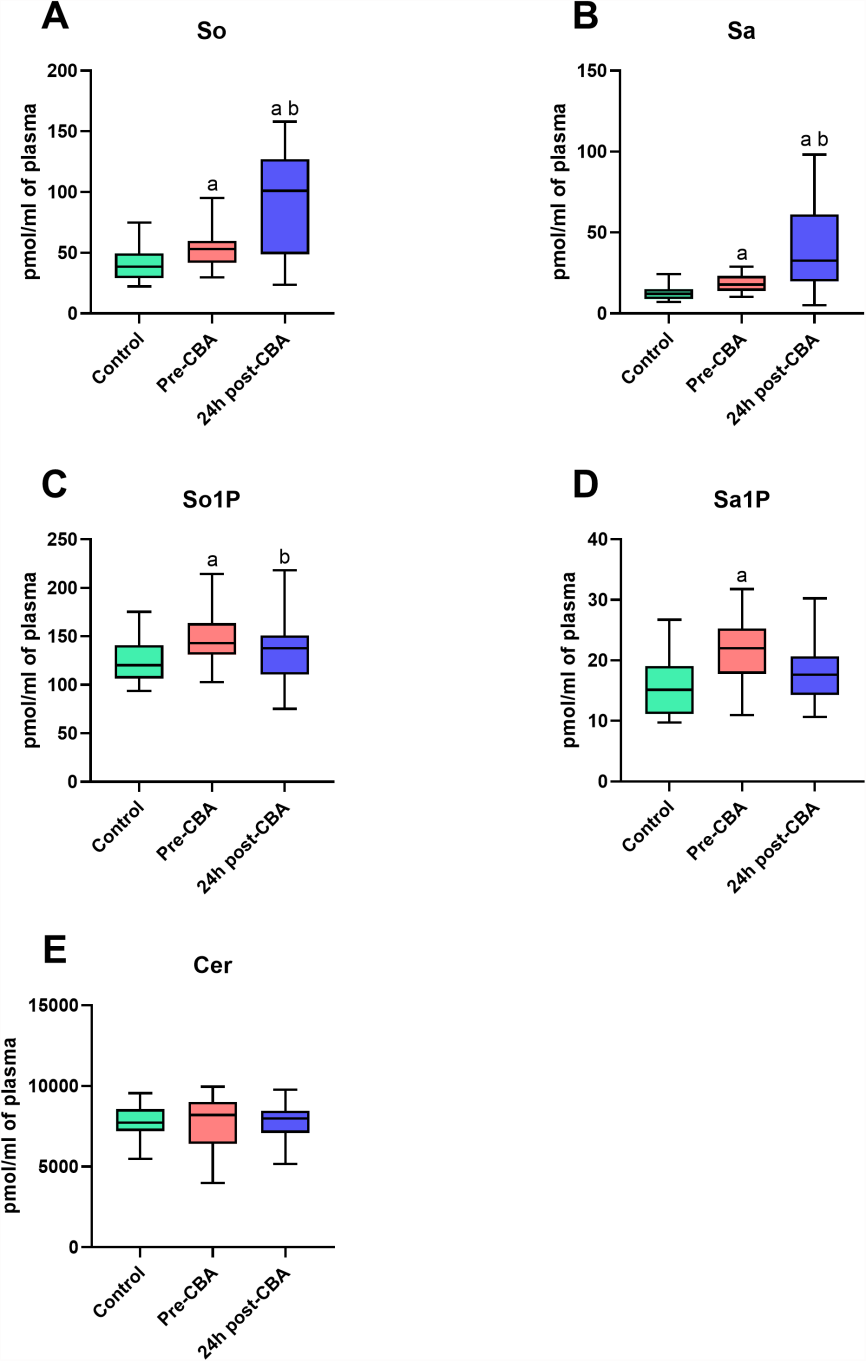
Sphingolipid profile in patients with atrial fibrillation before (pre-CBA) and 24 h after (24 h post-CBA) cryoballoon ablation (CBA) therapy and healthy volunteers (Control) in plasma fraction. Sphingosine (So), sphinganine (Sa), sphingosine-1-phosphate (So1P), sphinganine-1-phosphate (Sa1P), and ceramide (Cer). Data based on 20 independent measurements for the Control group and 29 independent measurements for both the Pre-CBA and 24 h post-CBA groups. The statistically significant difference was considered as a p-value < 0.05.

In plasma fraction, we observed significant increases in So, Sa, So1P, and Sa1P concentrations in AF-patients before cryoablation than in the healthy volunteers (So: +24.6%; Sa: +33.5%; So1P: +22.3%; Sa1P: +34.3%; p < 0.05; Fig 2A,2B,2C and 2D). The content of plasma So and Sa was also elevated in AF-patients 24 h after CBA therapy in relation to the individuals from Control and Pre-CBA groups (So: +119.6% and +76.2%; Sa: +195.0% and +121.0%, respectively; p < 0.05; Fig 2A and 2B). Furthermore, plasma So1P level was decreased 24 h after cryoballoon ablation in patients with AF than in the level before treatment (So1P: −11.2%; p < 0.05; Fig 2C).

We observed significant differences in the AUC values of So, Sa, So1P, and Sa1P concentrations in patients with AF before cryoablation in relation to the individuals from the Control group (So: 0.73; Sa: 0.78; So1P: 0.76; Sa1P: 0.79; p < 0.05; Table 2). Moreover, the AUC values of So, Sa, and So1P contents in patients 24 h after CBA compared to the individuals from Control or/and Pre-CBA were relevant changed (So: 0.83 and 0.76; Sa: 0.88 and 0.79; Sa1P: 0.68, respectively; p < 0.05; Table 2).

**Table 2:** The analysis of AUC values of sphingolipid concentrations of patients with atrial fibrillation (before and 24 h after cryoballoon ablation (CBA) therapy) and control individuals in plasma fraction.

	The values of Area Under the ROC Curve, 95% Confidence Interval of AUC, and *p*-value		
	Pre-CBA vs. Control	24 h post-CBA vs. Control	24 h post-CBA vs. Pre-CBA
So	0.73 0.58–0.88 0.0074	0.83 0.71–0.94 <0.0001	0.76 0.62–0.89 0.0006
Sa	0.78 0.64–0.92 0.0011	0.88 0.79–0.98 <0.0001	0.79 0.67–0.91 < 0.0001
So1P	0.76 0.62–0.90 0.0030	0.62 0.46–0.77 0.1577	0.65 0.51–0.79 0.0516
Sa1P	0.79 0.65–0.92 0.0008	0.66 0.50–0.81 0.0563	0.68 0.54–0.82 0.0166
Cer	0.55 0.38–0.72 0.5759	0.50 0.34–0.67 0.9707	0.57 0.42–0.73 0.3379

Sphingosine (So), sphinganine (Sa), sphingosine-1-phosphate (So1P), sphinganine-1-phosphate (Sa1P), and ceramide (Cer). The values of the Area Under the ROC Curve and 95% Confidence Interval of AUC are expressed in the range 0–1. Data based on 20 independent measurements for the Control group and 29 independent measurements for both the Pre-CBA and 24 h post-CBA groups. The statistically significant difference was considered as a p-value < 0.05.

### The comparison of sphingolipid concentrations in plasma very low-density lipoprotein cholesterol (VLDL-C), low-density lipoprotein cholesterol (LDL-C) fraction, and high-density lipoprotein cholesterol (HDL-C) fraction of patients with atrial fibrillation (before and 24 h after cryoballoon ablation (CBA) therapy) and control individuals

**Fig 3:**
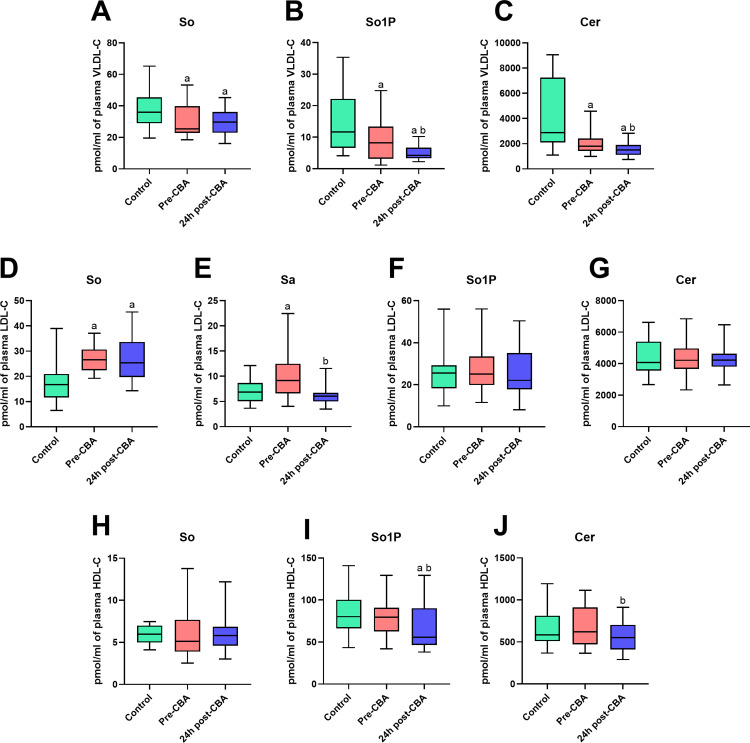
Sphingolipid profile in patients with atrial fibrillation before (pre-CBA) and 24 h after (24 h post-CBA) cryoballoon ablation (CBA) therapy and healthy volunteers (Control) in plasma very low-density lipoprotein cholesterol (VLDL-C) fraction, low-density lipoprotein cholesterol (LDL-C) fraction, and high-density lipoprotein cholesterol (HDL-C) fraction. Sphingosine (So), sphinganine (Sa), sphingosine-1-phosphate (So1P), sphinganine-1-phosphate (Sa1P) and ceramide (Cer). Data based on 20 independent measurements for the Control group and 29 independent measurements for both the Pre-CBA and 24 h post-CBA groups. The statistically significant difference was considered as a p-value < 0.05.

In VLDL-C fraction, we noticed significant decreases in So, So1P, and Cer concentrations in AF-patients before cryoablation than in the healthy volunteers (So: −19.5%; So1P: −48.7%; Cer: −43.1%; p < 0.05; Fig 3A–3C). In addition, the CBA treatment caused a reduction in the content of So, So1P, and Cer in VLDL-C fraction compared to the Control or/and Pre-CBA groups (So: −21.4%; So1P: −65.7% and −33.2%; Cer: −65.0% and −38.6, respectively; p < 0.05; Fig 3A–3C).

In LDL-C lipoprotein fraction, the level of So and Sa was higher in AF-patients before CBA in relation to the healthy volunteers (So: +49.6%; Sa: +46.0%; p < 0.05; Fig 3D and 3E). Importantly, the So content was increased 24 h after CBA in comparison with the individuals from the Control group (So: +47.2%; p < 0.05; Fig 3D) and the Sa content was decreased 24 h after CBA procedure compared to the patients from Pre-CBA group (Sa: −32.3%; p < 0.05; Fig 3E).

In HDL-C fraction, the So1P level was decreased only in patients from 24 h post-CBA group in comparison with healthy volunteers and patients with AF before cryoablation therapy (So1P: −20.2% and −15.1%, respectively; p < 0.05; Fig 3I). Moreover, the Cer content was reduced 24 h after CBA in relation to the individuals from Control group (Cer: −18.1%; p < 0.05; Fig 3J).

The AUC values of So1P and Cer in VLDL-C in patients before CBA comparison with individuals from the Control group were significantly changed (So1P: 0.67; CER: 0.77; p < 0.05; Table 3). We also observed significant differences in the AUC values of So, So1P, and Cer concentrations in patients with AF 24 h after cryoablation in relation to the individuals from Control or/and Pre-CBA groups (So: 0.67; So1P: 0.88 and 0.67; Cer: 0.90 and 0.70, respectively; p < 0.05; Table 3).

**Table 3:** The analysis of AUC values of sphingolipid of patients with atrial fibrillation (before and 24 h after cryoballoon ablation (CBA) therapy) and control individuals concentrations in plasma very low-density lipoprotein cholesterol (VLDL-C) fraction.

	The values of Area Under the ROC Curve, 95% Confidence Interval of AUC, and *p*-value		
	Pre-CBA vs. Control	24 h post-CBA vs. Control	24 h post-CBA vs. Pre-CBA
So	0.65 0.48–0.81 0.0838	0.67 0.51–0.83 0.0409	0.50 0.35–0.65 0.9938
So1P	0.67 0.52–0.83 0.0430	0.88 0.78–0.98 <0.0001	0.67 0.51–0.82 0.0344
Cer	0.77 0.38–0.72 0.5759	0.90 0.81–1.00 <0.0001	0.70 0.56–0.84 0.0099

Sphingosine (So), sphingosine-1-phosphate (So1P), and ceramide (Cer). The values of Area Under the ROC Curve and 95% Confidence Interval of AUC are expressed in the range 0–1. Data based on 20 independent measurements for the Control group and 29 independent measurements for both the Pre-CBA and 24 h post-CBA groups. The statistically significant difference was considered as a p-value < 0.05.

The AUC values of So and Sa in LDL-C in patients before CBA comparison with individuals from the Control group were significantly changed (So: 0.82; Sa: 0.70; p < 0.05; Table 4). We also observed pronounced differences in the AUC values of So content in patients with AF from the 24 h post-CBA group in relation to the healthy volunteers (So: 0.76; p < 0.05; Table 4). Furthermore, the AUC values of Sa in AF-patients 24 h after CBA than in before CBA therapy were different (Sa: 0.79; p < 0.05; Table 4).

**Table 4:** The analysis of AUC values of sphingolipid concentrations of patients with atrial fibrillation (before and 24 h after cryoballoon ablation (CBA) therapy) and control individuals in plasma low-density lipoprotein cholesterol (LDL-C) fraction.

	The values of Area Under the ROC Curve, 95% Confidence Interval of AUC, and *p*-value		
	Pre-CBA vs. Control	24 h post-CBA vs. Control	24 h post-CBA vs. Pre-CBA
So	0.82 0.67–0.97 0.0002	0.76 0.62–0.90 0.0019	0.50 0.35–0.66 0.9873
Sa	0.70 0.56–0.85 0.0168	0.59 0.41–0.76 0.3116	0.79 0.67–0.91 0.0002
So1P	0.54 0.38–0.71 0.6111	0.62 0.36–0.69 0.7758	0.55 0.40–0.70 0.4889
Cer	0.52 0.35–0.70 0.7857	0.52 0.34–0.69 0.8388	0.51 0.36–0.67 0.8606

Sphingosine (So), sphinganine (Sa), sphingosine-1-phosphate (So1P), and ceramide (Cer). The values of Area Under the ROC Curve and 95% Confidence Interval of AUC are expressed in the range 0–1. Data based on 20 independent measurements for the Control group and 29 independent measurements for both the Pre-CBA and 24 h post-CBA groups. The statistically significant difference was considered as a p-value < 0.05.

In the HDL-C lipoprotein pool, the AUC values of So1P in AF-patients from the 24 h-post CBA group compared to the individuals from the Control and Pre-CBA groups were markedly changed (So1P: 0.72 and 0.67, respectively; p < 0.05; Table 5).

**Table 5:** The analysis of AUC values of sphingolipid concentrations of patients with atrial fibrillation (before and 24 h after cryoballoon ablation (CBA) therapy) and control individuals in plasma high-density lipoprotein cholesterol (HDL-C) fraction.

	The values of Area Under the ROC Curve, 95% Confidence Interval of AUC, and *p*-value		
	Pre-CBA vs. Control	24 h post-CBA vs. Control	24 h post-CBA vs. Pre-CBA
So	0.57 0.40–0.73 0.4416	0.57 0.41–0.73 0.4170	0.50 0.35–0.65 0.9752
So1P	0.56 0.39–0.72 0.5151	0.72 0.57–0.86 0.0098	0.67 0.52–0.81 0.0289
Cer	0.53 0.36–0.69 0.7448	0.61 0.45–0.77 0.2072	0.64 0.50–0.78 0.0677

Sphingosine (So), sphingosine-1-phosphate (So1P), and ceramide (Cer). The values of Area Under the ROC Curve and 95% Confidence Interval of AUC are expressed in the range 0–1. Data based on 20 independent measurements for the Control group and 29 independent measurements for both the Pre-CBA and 24 h post-CBA groups. The statistically significant difference was considered as a p-value < 0.05.

## Discussion

In recent years, significant progress has been made in understanding the mechanisms of AF development, indicating a complex interplay of triggers. There is a large consensus regarding the implication of lipid abnormalities on AF incidence as an important risk factor. Numerous evidence indicates that changes in the lipid profile most commonly include a reduced HDL-C level as well as an increased concentration of LDL-C and TAG particles, which contributes to the picture of dyslipidemia often accompanying obesity [5]. However, we should bear in mind that recent studies provided inconsistent data regarding the role of dyslipidemia in the AF onset, and the exact association between plasma lipid levels and the incidence of AF has not been fully clarified [32,33]. Moreover, as some research points out, even the improvement of lipid profile parameters (e.g., LDL-C level treated to goal) leaves a significant risk for CVDs [34]. Hence, the question arises whether other lipid profile parameters can be used as predictors of cardiac event occurrence or the effectiveness of the treatment method used. This applies in particular to the group of patients with AF due to increasing prevalence of this type of arrythmia worldwide. Therefore, the present study aims to fill this knowledge gap by examining a wide panel of 9 Apo as well as plasma concentrations of selected sphingolipids and their distribution in individual lipoprotein fractions in patients with AF undergoing cryoballoon ablation therapy.

Apo have been of increasing interest to researchers for some time due to their diverse biological functions and association with multifarious disorders. They are mostly synthesized in the liver and have been shown to play a significant role in lipid metabolism, transport, and stabilization of lipoproteins, where they constitute an essential structural component [35]. Furthermore, accumulating evidence suggests that alterations in Apo level are closely related to an increased risk of various diseases, including different CVDs, however, the association with the risk of AF incidence has been inconsistent [36]. In our study, we observed slightly reduced concentrations of both Apo-A1 and Apo-A2 in the group of patients with AF compared to control subjects. Both Apo are critical structural components of HDL-C particles, however, they differ significantly in biological function [11,37]. Apo-A1 is widely known for modulating cholesterol trafficking through the stabilization of the ATP-binding cassette transporter 1 (ABCA1) localized at the cell membrane of hepatocytes and enterocytes, and activation of lecithin-cholesterol acyltransferase (LCAT), which collectively participate in initiating the reverse cholesterol transport [38]. In turn, clinical studies indicated that Apo-A2 serves as LPL activity regulator mainly via modification of HDL-C proteome [39]. Evidence for the role of Apo-A2 in TAG catabolism was supported by studies conducted in mice overexpressing the Apo-A2 gene, which demonstrated a positive correlation between plasma Apo-A2 and TAG levels [39]. Taking into account the roles played by the Apo-A1 and Apo-A2 molecules, it is believed that they have the opposite effect in CVDs. In particular, Apo-A-1 is considered to perform an atheroprotective function, whereas Apo-A2 is characterized as a proatherogenic molecule. However, the results obtained from patients with AF are still controversial, and recent studies have shown that either Apo-A1 and Apo-A2 concentrations were markedly lower in AF patients in comparison with healthy participants [40,41]. We assume that the observed in our study lack of changes in the levels of Apo-A1 and Apo-A2 in patients suffering from AF is probably due to the relevant heterogeneity of the examined group and the use of lipid-lowering medications. Interestingly, in the present study, the patients after cryotherapy presented substantially decreased plasma concentrations of both Apo-A1 and Apo-A2, thus our results suggest that the ablation procedure influences the composition of HDL-C particles. Furthermore, our results indicated that the patients 24 h after the CBA procedure demonstrated a significant reduction in the Apo-B level in comparison with the pre-cryoablation concentration. This is worth emphasizing since Apo-B constitutes a principal structural component of VLDL-C, IDL-C, and LDL-C, thereby its concentration reflects the total quantity of circulating atherogenic molecules [5]. Moreover, a lot of research indicated a direct correlation between the increased level of Apo-B and raised incidence of CAD suggesting that Apo-B may serve as a sensitive and adequate indicator for assessing cardiovascular risk [6,42]. However, for patients with AF, discrepancies re-emerge as studies conducted by Ding et al. showed that elevated Apo-B levels were not associated with the onset of AF [43]. These results suggest that at least part of the etiology of AF differs from that of atherosclerotic heart disease and that an imbalance between proatherogenic and antiatherosclerotic molecules is not the major underlying mechanism for the development of AF. Accordingly, in our study, we noticed a pronounced decrease in Apo-C1 levels in patients 24 h after the cryoablation compared to patients before the procedure. Apo-C1 is considered as an important regulator of lipid metabolism since it appears to have a broader effect on different classes of lipoproteins [44]. Firstly, Apo-C1 takes part in the metabolism of TRL, inhibiting their binding to specific receptors mainly by modulating LPL activity, which can lead to increased plasma TAG concentrations [45]. The second important function of Apo-C1 is participation in HDL-C remodeling by reducing the cholesteryl ester transfer protein (CETP) activity [46]. A substantial amount of studies demonstrated that lower CETP levels favor the formation of HDL-C, a particle with well-documented atheroprotective functions [44]. Based on the above properties of Apo-C1, we can hypothesize that it may have distinct functions depending on its distribution among lipoproteins (with a proatherogenic effect in combination with VLDL-C and an antiatherosclerotic effect in combination with HDL-C particles), therefore a clear influence of Apo-C1 on cardiometabolic risk is difficult to establish and further research needs to be done, especially in patients with AF. Concomitantly, Apo-E is encoded by the same cluster of genes as Apo-C1 and therefore belongs to the group of apolipoproteins that perform crucial functions in controlling plasma lipid levels with subsequent implications in cardiovascular health [47]. It is well-established that Apo-E exhibits anti-atherogenic activity, which is largely due to its role in lowering plasma cholesterol concentrations by favoring the uptake of TRL from circulation [48]. Nevertheless, Tsai et al. reported that the Apo-E gene upregulation may be associated with the occurrence of AF [49], while another study demonstrated a correlation between Apo-E polymorphism and the AF incidence in the Alzheimer’s disease population [50]. In our research, we did not notice any pronounced alterations in the plasma concentration of Apo-E in the group of patients with AF, however, the cryoablation caused a noticeable decrease in this Apo level. Collectively, these findings may not fully explain the effect of Apo-E on AF, hence taking into account the multifactorial nature of this cardiac arrhythmia, we can conclude that Apo-E may be involved in the development of this disorder through other mechanisms. In this study, we also reported that the CBA procedure caused a substantial reduction of Apo-D concentration in comparison with healthy participants as well as compared to the group of patients with AF. Apo-D was initially considered to be only a lipophilic molecules transporter, however, data provided from numerous animal and human studies have revealed new beneficial properties of this Apo, such as antioxidant and anti-inflammatory activity, which can contribute to the cardioprotective effects [15,51,52]. Similarly, recent studies have shown that Apo-J also has an antioxidant effect due to its association with HDL-C particles [53]. However, a growing body of evidence suggests that Apo-J has a more dualistic nature resulting from its distribution among various lipoproteins and plasma [18]. These conclusions were drawn based on the detection of a positive correlation between the elevated level of circulating Apo-J and obesity, type 2 diabetes, inflammation, and, importantly, various CVDs including atherosclerosis, CAD, and myocardial infarction. Probably this is related to a decrease in the content of Apo-J in the HDL-C molecule and its proteome remodeling, rather than to the increased production of this Apo [18,54]. In contrast, our data demonstrated markedly reduced Apo-J concentration in patients before the cryoablation compared to the control subjects, whereas more interestingly, cryotherapy in patients with AF reduced the concentration of Apo-J to an even greater extent compared to patients before the CBA procedure. Thus, it is still unclear whether Apo-J has a positive or negative impact on the cardiovascular system. In addition, in the present study, we perform the receiver operating characteristic (ROC) analysis for plasma Apo distribution to find potential biomarkers for predicting AF incidence as well as to evaluate the specific and sensitive biomarkers of the treatment effectiveness in AF patients undergoing cryoablation. Our current analyses reveal that none of the Apo is very accurate in predicting the onset of AF, but, notably, it shows that plasma concentrations of Apo-J can be used as a sensitive non-invasive short-term biomarker of cryotherapy success (confirming maintenance of sinus rhythm) in patients with AF. Unfortunately, although the short-term success rate of CBA procedure is high, recurrences of AF do occur among patients during long-term follow-up. Therefore, it is necessary to conduct future research in a larger study population with a longer follow-up period to certainly confirm the possibility of using Apo-J as a predictive plasma biomarker of the long-term effectiveness of cryoablation (no recurrence of AF), which in the future could be used as a non-invasive marker in clinical practice. This finding seems to be extremely important since there are currently no plasma biomarkers confirming the catheter ablation efficacy. In general, the inconsistency in our results and those of other studies may be partly explained by methodological differences, such as variations in demographic characteristics of the study population, sample size, and the use of cardiovascular drugs, hence future research will be needed to extend our knowledge about Apo distribution in AF patients.

Many investigations in recent years have shed new light on the bioactive lipid species, which form the sphingolipid class. They constitute a very diverse group of lipids that perform various functions related to the construction of cell membranes, transmitting intracellular signals, and regulating cell growth processes [21]. Moreover, the constantly rising number of studies highlight the principal functions of sphingolipids in the pathophysiology of distinct cardiometabolic diseases, such as hypertension, myocardial infarction, and heart failure [55]. Therefore, it seems reasonable to assume that alternations in plasma sphingolipids concentrations can be used as prognostic and diagnostic markers of various cardiovascular events. To the best of our knowledge, the herein study is the first, which examines the total plasma concentration of the sphingolipids as well as their content in selected lipoprotein fractions in AF-patients before and 24 h after cryoballoon ablation therapy. Our results demonstrated substantially elevated plasma concentrations of So and Sa in patients with AF in both Pre-CBA and 24 h post-CBA groups compared to healthy participants. Interestingly, we also noticed that in AF patients undergoing CBA procedure the levels of the above-mentioned sphingolipids were enhanced to a much greater extent in comparison with the group of patients before the cryotherapy. Similar results were obtained by Egom et al., who showed elevated concentrations of Sa, So, and So1P in patients after transient cardiac ischaemia following the percutaneous coronary intervention [56]. One of possible explanation of these alternations is the activation of the sphingolipids recycling pathway, in which Cer is deacylated to form So, as a result of stimulation by pro-inflammatory cytokines [21]. This is consistent with the fact that the cryoablation procedure is associated with increased inflammatory response and myocardial injury in the post-procedure period, which we have also shown in our previous publication [24,57]. This assumption is also in line with the Cer concentration, which is slightly lower in patients 24 hours after cryotherapy in comparison with the Pre-CBA group. Concomitantly, in our study, we observed that patients with AF presented substantially increased plasma levels of So1P and Sa1P in comparison with controls. This is an intriguing finding as So1P has been known for many years to play a pivotal role in cellular processes, such as proliferation, differentiation, migration, and suppression of apoptosis [58,59]. Moreover, Karliner et al. demonstrated that the treatment with exogenous So1P of both cultured cardiac myocytes undergoing hypoxia as well as isolated hearts prior to ischemia or at the beginning of reperfusion has a prosurvival effect, which is considered as cardioprotective action [60]. In contrast, another study found a positive correlation between serum So1P levels and the incidence of CAD, where So1P was observed to increase with disease severity, which additionally indicates that it may serve as a strong predictor of obstructive CAD [61]. This is in agreement with our results and we suspect that the likely mechanism underlying these changes is the involvement of oxidative stress in the AF pathogenesis [62]. It has been proven that reactive oxygen species (ROS) activate the sphingosine kinase 1 (SPHK1) enzyme, which participates in the phosphorylation of So to generate So1P, and hence in patients with AF, we noticed markedly elevated plasma So1P concentration [63]. The precise regulatory mechanism of So1P in AF is not completely explained, however, based on the above data, it can be assumed that So1P may be involved in myocardial remodeling and its exact role also depends on the stage of the disease. Taken together, pronounced changes in sphingolipid levels in AF patients undergoing CBA treatment reflect the complex interconversion pathways and dynamic metabolism of these sphingolipids in the circulatory system. Thus, in order to better understand the role of distinct sphingolipid molecules in the etiology of AF, we examined in our study their content in selected lipoprotein fractions, which may help to create new therapeutic options in treating AF or provide new data on the effectiveness of antiarrhythmic therapy. Lipoprotein particles are the main carriers of sphingolipids in the plasma, and therefore it should be considered whether modifications of sphingolipids content may potentially alter the function and properties of lipoproteins, especially in AF. With regard to the VLDL-C particles, we reported substantially reduced levels of So, So1P, and Cer in patients with AF from both Pre-CBA and 24 h post-CBA groups in comparison with the healthy individuals, whereas in the LDL-C lipoprotein pool, the content of plasma So and Sa was elevated in subjects with AF in relation to the controls. Sa, So, and Cer are considered as proatherosclerotic lipid mediators since circulating concentrations of these sphingolipids have been reported to be associated with an increased risk of atherosclerosis [64]. Therefore, their increased content within LDL-C may partly be responsible for its harmful and proatherogenic properties, which can contribute to the pathogenesis of AF [3]. Surprisingly, the latter studies show that the LDL-bound ceramides were negatively associated with CVDs occurrence, which is unexpected since plasma and serum Cer concentrations are known to be higher in patients with CVDs [64]. In the case of HDL-C particles, the content of Cer was markedly reduced in the group of post-ablation patients, as compared with subjects from Pre-CBA group. These observations are also puzzling since previous studies, similar to the case of LDL-C, have shown an inverse relationship between the level of Cer present in the HDL-C fraction and the occurrence of atherosclerosis and ischemic heart disease (IHD) [65,66]. Additionally, in our study, we noticed that cryotherapy resulted in a significant decrease in So1P levels in both VLDL-C and HDL-C fractions compared to the pre-cryoballoon ablation state. Unlike So and Cer, So1P is reported as a potent bioactive lipid with beneficial anti-inflammatory and vasoprotective activity [67]. Recent studies have shown that So1P is predominantly bound to HDL-C molecules, and therefore changes in its concentration lead to significant alternations of the HDL-C composition [65]. As a result, HDL-C functions may be disrupted and thus this may reduce the ability of HDL-C to induce the outflow of sphingolipids accumulated in peripheral tissues [65]. Hence, due to intramolecular changes in lipid content, HDL-C may lose its protective functions and even become a proatherogenic agent. This hypothesis is confirmed by research conducted by Argraves et al., which proved that the So1P level in HDL-C is inversely related to the IHD occurrence [66]. Therefore, the discrepancies observed in our study raise numerous questions that require further research in the future to elucidate the carrier-dependent bioactivity of various sphingolipids and to evaluate the exact mechanism of the observed alternations in the group of patients with AF undergoing cryoablation.

There are several potential limitations to the present study that are worth considering. First, this was a single-center case-control study with a relatively small group of participants. Secondly, the controls and the AF group differed substantially from each other, since it was not possible to qualify the elderly and obese subjects to the group of healthy volunteers, due to the fact that these features are important risk factors for the development of various CVDs. Third, medication characteristics and occurring comorbidities of AF patients and controls were not well matched. The use of lipid-lowering drugs in patients with AF, but not in healthy subjects, could influence the contents of lipids in the plasma lipoproteins. In addition, several potential confounding factors, including genetic factors, family history, and lifestyle may have also affected the present outcomes. Nevertheless, this study provides us a new perspective in the search for potential predictive markers for the early detection of AF incidence and biomarkers indicating the efficacy of the applied cryoablation procedure. However, to confidently confirm the potential of apolipoproteins and sphingolipids as biomarkers of the long-term cryoablation effectiveness in patients with AF, changes in their plasma concentrations should be assessed in a larger study population during the follow-up period.

## Conclusions

To improve patient care and management of AF, a better understanding of its pathophysiology is needed, as actual antiarrhythmic therapies have limited effectiveness and off-target effects. Therefore, in the present study, we investigated for the first time the behavior of plasma Apo and sphingolipids in patients with AF undergoing the CBA procedure. Unexpectedly, we did not observe any significant associations between AF and plasma Apo concentrations, however, cryotherapy profoundly alters the Apo profiles in relation to the pre-cryoballoon ablation group and healthy volunteers. Additionally, our analyses of the AUC values reveal that none of the Apo is very accurate in predicting AF development, but, notably, it shows that plasma concentrations of Apo-J can be used as a potential short-term biomarker indicating cryotherapy success in patients with AF. Furthermore, our results also demonstrated pronounced abnormalities in the sphingolipid composition in both plasma and individual lipoprotein fractions in AF patients before treatment and 24 h after the CBA. Due to the multidirectional action of sphingolipids, the impairment of their metabolism might be one of the risk factors for the development of various cardiometabolic diseases, including AF. However, it is still unclear whether the changes observed in the present study are the basis of AF or they constitute a consequence of the arrhythmia itself. Therefore, taking into account the limitations of our study, further research is required to confirm whether Apo and sphingolipids could be a useful biomarkers in predicting the AF onset and assessing the efficacy of the CBA therapy.

## Supporting information

S1 Data Apolipoproteins profile. Apolipoprotein A1, A2, B, C1, C3, D, E, H and J (Apo-A1, -A2, -B, -C1, -C3, -D, -E, -H and -J).
(XLS)

S2 Data Sphingolipids profile. Sphingosine (So), sphinganine (Sa), sphingosine-1-phosphate (So1P), sphinganine-1-phosphate (Sa1P), and ceramide (Cer).
(XLS)

## Abbreviations

AF: atrial fibrillation
CVD: cardiovascular diseases
LV: left ventricle
CBA: balloon-based cryoablation
pre-CBA: group of AF patients before the cryoablation
24 h post-CBA: group of AF patients 24 h after cryoablation
HPLC: high-performance liquid chromatography
Apo-A1, -A2, -B, -C1, -C3, -D, -E, -H and -J: apolipoprotein A1, A2, B, C1, C3, D, E, H and J
VLDL-C: very low-density lipoprotein cholesterol
LDL-C: low-density lipoprotein cholesterol
HDL-C: high-density lipoprotein cholesterol
So: sphingosine
Sa: sphinganine
So1P: sphingosine-1-phosphate
Sa1P: sphinganine-1-phosphate
Cer: ceramide
